# PtBGL: a cost-effective alternative to GUS reporter with applications in plant imprint dyeing

**DOI:** 10.3389/fpls.2025.1705524

**Published:** 2026-01-06

**Authors:** Yuyao Su, Yitong Li, Jiali Zhong, Yudan Wang, Jiayin Wang, Xiaopeng Li, Jia Li, Yao Xiao

**Affiliations:** Guangdong Provincial Key Laboratory of Plant Adaptation and Molecular Design, School of Life Sciences, Guangzhou University, Guangzhou, China

**Keywords:** cheap substrate, gene expression, imprint dyeing, PtBGL, reporter gene

## Abstract

Reporter gene systems are essential tools for monitoring gene expression and transformation efficiency in plant research. The widely used GUS system provides strong, stable, and well-localized signals but requires expensive substrates. Here, we present an alternative based on *Persicaria tinctoria* β-glucosidase (PtBGL) and its natural substrate indican, which is a low-cost indoxyl glucoside extractable from heat-treated indigo plants. When expressed in *Arabidopsis thaliana* and *Nicotiana benthamiana*, PtBGL localizes to plastids and converts indican into a blue indigo pigment visible to the naked eye. Comparative assays in 13 of the 14 tested plant species revealed no detectable background activity from endogenous β-glucosidases at the examined developmental stage. The PtBGL and indican system enables cost-effective transgenic screening, gene expression analysis, and practical applications such as plant-based fabric imprinting.

## Introduction

The β-glucuronidase (GUS) reporter system has been widely used in plant research since 1987 to evaluate transformation efficiency, analyze gene expression patterns, and characterize promoter activity (Jefferson et al., 1987). Encoded by the *uidA* gene from *Escherichia coli*, GUS catalyzes the hydrolysis of 5-bromo-4-chloro-3-indolyl-β-D-glucuronide (X-gluc) into indoxyl derivatives, which are then oxidized and dimerized to form an insoluble blue indigoid precipitate. Compared with alternative reporters such as fluorescent proteins (e.g., GFP, RFP) and luciferase, GUS assays provide stable signals with strong contrast, without requiring specialized imaging equipment or raising concerns about autofluorescence (De Ruijter et al., 2003). In addition, GUS activity persists in fixed tissues, allowing long-term and *post hoc* analyses, and offers robust, well-localized visualization of gene expression at both tissue and cellular levels. However, the high cost of substrates like X-gluc and 4-methylumbelliferyl-β-D-glucuronide (MUG) limits the use of GUS assays in large-scale or high-throughput studies, especially for large plants (Mudunkothge et al., 2023).

Other enzymes, such as β-galactosidase (e.g., LacZ) and β-glucosidase, have also been used as reporters through cleavage of glycoside substrates to yield colored precipitates. LacZ hydrolyzes 5-bromo-4-chloro-3-indolyl-β-D-galactopyranoside (X-gal), a cheaper substrate than X-gluc. However, its application in higher organisms is often hindered by endogenous β-galactosidase activity (Teeri et al., 1989). The synthetic β-glucosidase SYNbglA, engineered from *Caldocellum saccharolyticum*, has been employed in mammalian reporter systems with costly substrates like o-nitrophenyl-β-D-glucopyranoside (ONP-glu) and 5-bromo-4-chloro-3-indolyl-β-D-glucopyranoside (BCI-glu) (McCutcheon et al., 2010). Similarly, the mutant glucosidase cpGluT, exhibiting activity toward X-gal, MUG, and indoxyl-β-D-glucoside (indican), facilitates efficient recombinant plasmid detection during cloning procedures (Cheong et al., 2012). The application of glucosidases as reporters in plant systems remains unexplored.

**Figure 1:**
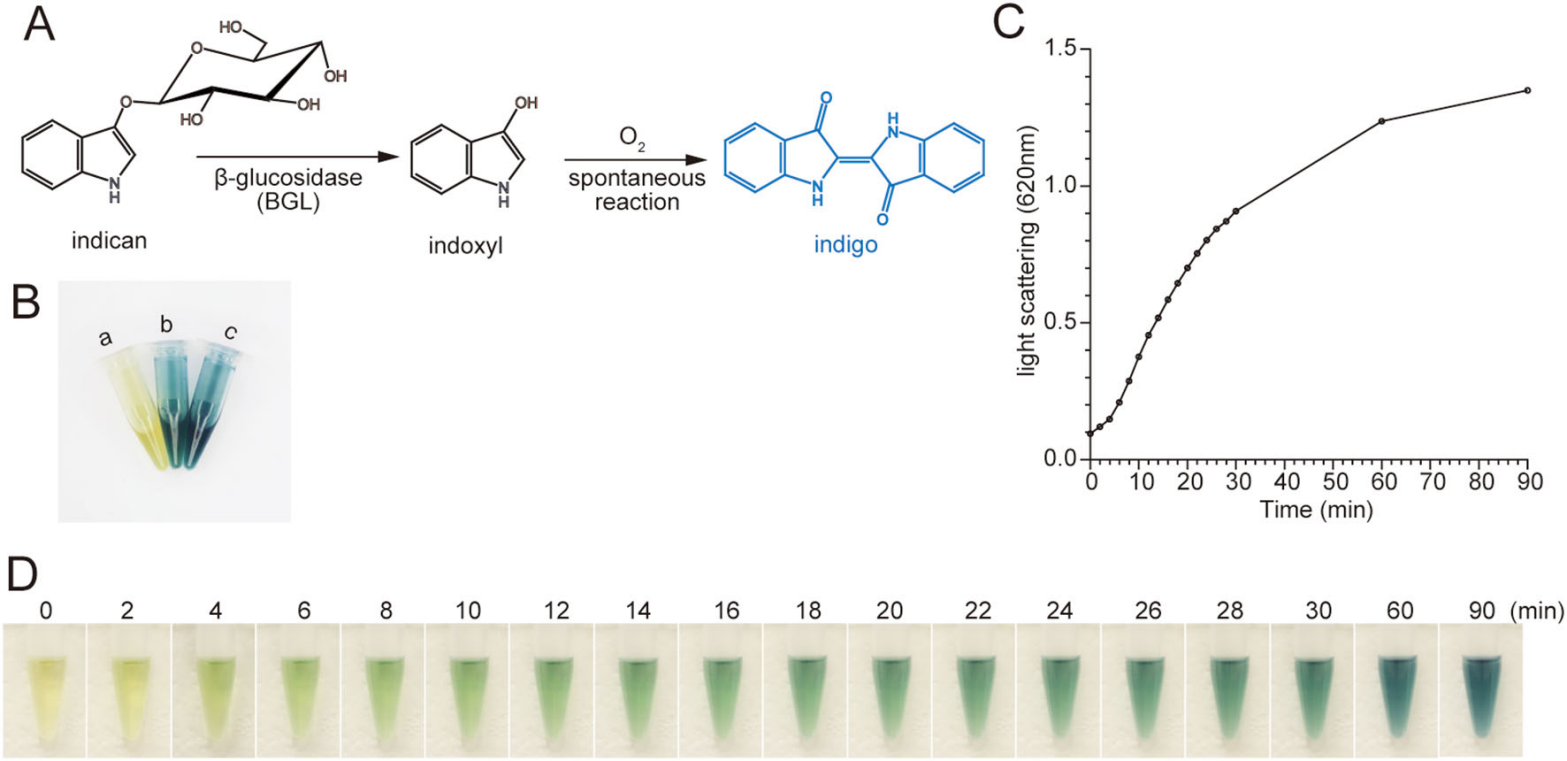
Rapid indigo production catalyzed by *Persicaria tinctoria* β-glucosidase (PtBGL) in tobacco leaf lysates. **(A)** Schematic of the enzymatic reaction: PtBGL hydrolyzes indoxyl-β-D-glucoside (indican) to release indoxyl, which is spontaneously oxidized in the presence of molecular oxygen and subsequently dimerizes to form insoluble indigo. **(B)** Visible blue coloration in tobacco lysates 2 h after indican addition. a, empty vector control; b, full-length PtBGL; c, ΔTP-PtBGL. **(C)** Time course of indigo particle formation monitored by light scattering at 620 nm. **(D)** Corresponding visual observations at the indicated time points. Panels **(C, D)** show representative results from three independent experiments with similar outcomes.

*Persicaria tinctoria* (syn. *Polygonum tinctorium)* β-glucosidase (PtBGL) hydrolyzes indican, a naturally occurring and abundant substrate found in indigo-producing plants (Minami et al., 1996; Inoue et al., 2020). Indican is substantially less expensive and can be extracted as crude material at negligible cost (Jiang et al., 2022; Hu et al., 2023). The enzymatic hydrolysis releases indoxyl, which is oxidized to form insoluble indigo pigment (**Figure 1A**). Therefore, PtBGL represents a promising alternative to the GUS reporter system in plants. Still, whether heterologously expressed PtBGL retains activity and whether it suffers from background interference similar to LacZ–X-gal have not been investigated.

Here, we demonstrate that the PtBGL–indican system exhibits high specificity with minimal background interference across multiple plant species. PtBGL remains enzymatically active when heterologously expressed in both *Arabidopsis thaliana* and *Nicotiana benthamiana*, serving effectively as a marker for genetic screening and gene expression pattern analysis. Beyond reporting, we explore PtBGL’s potential in plant-based dyeing by employing simple extraction methods for crude indican and applying it for durable fabric imprinting. This system provides a cost-effective, scalable alternative to conventional reporters and introduces a sustainable, fade-resistant dyeing technology.

## Materials and methods

### Plant growth conditions

All plants, except *Arabidopsis thaliana*, *Marchantia polymorpha*, *Persicaria tinctoria*, and *Solanum tuberosum*, were grown in a growth chamber (HiPoint FH-600) at 25°C under a 16 h light/8 h dark photoperiod on Murashige and Skoog (MS) medium. *Arabidopsis* and *Marchantia polymorpha* were cultivated in a growth chamber (Xunon PFG850) at 22°C under the same photoperiod, on MS medium and half-strength Gamborg’s B5 medium, respectively. *Persicaria tinctoria* and *Solanum tuberosum* were grown in soil under the same photoperiod at 25°C.

*Arabidopsis* seeds were surface-sterilized in 1% (v/v) sodium hypochlorite solution for 7 min, followed by five washes with sterile water. The sterilized seeds were then vernalized in the dark at 4°C for 2 days before being sown on vertical plates containing MS medium supplemented with 0.05% (w/v) 2-(N-morpholino)ethanesulfonic acid (MES), 0.8% (w/v) agar, and 1% (w/v) sucrose. Seven-day-old seedlings were transplanted into soil for further growth and phenotype observation. Seedlings at other developmental stages were used for specific assays, including staining and genotyping, with precise ages detailed in the figure legends. The Columbia-0 (Col-0) ecotype was employed as the genetic background for transformation and served as the control in staining experiments.

### Cloning procedures and plant transformation

Total RNA was extracted from *Persicaria tinctoria* leaves using the RNAprep Pure Plant Kit (Vazyme, RC411-01), and first-strand cDNA was synthesized with the HiScript II 1st Strand cDNA Synthesis Kit (Yeasen, 11120ES60). Both the full-length and a truncated PtBGL lacking the N-terminal 28 amino acids corresponding to the chloroplast transit peptide were amplified by PCR and cloned into the pGGC000 entry vector. Using the GreenGate cloning system with pGGZ000 as the destination vector backbone (Lampropoulos et al., 2013), a Pro35S-driven C-terminally mCitrine-tagged PtBGL expression construct was generated and introduced into *Agrobacterium tumefaciens* strain AGL1. Additional expression vectors containing full-length or truncated PtBGL, with or without mCitrine tags, were constructed in the pFASTR-A-G backbone under the control of the Pro35S, ProUBQ10 (635 bp), or ProATHB8 (2011 bp) promoters.

BGL sequences from *Arabidopsis*, *Brassica rapa*, *Oryza sativa*, and *Marchantia polymorpha* were identified via NCBI BLASTP searches against the PtBGL protein sequence. The full-length coding sequences of these orthologs were amplified and cloned using the same procedure. The PCR products were seamlessly inserted into the *Nco*I–*Avr*II site of a pCAMBIA1301-mCitrine vector. The pCAMBIA1301-mCitrine vector was generated by replacing the GUS gene between the *Nco*I and *Eco*91I sites of pCAMBIA1301 with a linker (GSGAGAG) fused to N terminal of mCitrine via In-Fusion cloning method. The resulting constructs were transformed into *A. tumefaciens* strain GV3101. All primers used in this study are listed in **Supplementary Table 1**.

For transient expression assays in tobacco, *Agrobacterium* suspensions were adjusted to an OD600 of 0.4–0.6 or a defined concentration gradient in infiltration buffer (1% (w/v) sucrose, 150 μM acetosyringone, 10 mM magnesium chloride, 10 mM MES, pH 5.7) and infiltrated into tobacco leaves. *Arabidopsis* transformation was performed using the floral dip method. T1 and T2 seeds were preliminarily screened for mRuby fluorescence using the LUYOR-3415RG excitation light source. During T1 screening, a mixture of non-fluorescent or weakly fluorescent seeds was intentionally included to serve as negative controls in downstream staining assays.

### Protein alignment, structure prediction and docking

The amino acid sequence of PtBGL (BAA78708.1) was retrieved from the NCBI database. The catalytic domain (residues Lys35–Asn511) was modeled using AlphaFold3 (https://alphafoldserver.com). Molecular docking of indican (PubChem CID, 441564) to the predicted PtBGL structure was performed using AutoDock 4.2. The best binding pose exhibited a binding free energy of –5.42 kcal/mol. Docking poses were analyzed for key ligand-protein interactions, including hydrogen bonding and π-π stacking, and visualized using PyMOL v3.0.3.

Multiple sequence alignment of five β-glucosidases evaluated in catalytic assays was performed using Clustal Omega 1.2.2 in Geneious Prime v12.7. Default gap opening and extension penalties were applied. Conserved residues implicated in indican binding were identified based on docking results and annotated accordingly.

### Enzymatic hydrolysis of indican by PtBGL in plant crude extracts

Tobacco leaves transiently expressing PtBGL were harvested two days after Agrobacterium infiltration. 0.05 g of leaf tissue was homogenized in 2 mL water containing 1 × cOmplete protease inhibitor cocktail (Roche, 04693132001) using a bead mill, and the homogenate was centrifuged at 12,000 × g for 3 min to obtain the supernatant. Indican (Sangon, A425761) was added to the supernatant to a final concentration of 110 μg/mL, after which the reaction mixture was aliquoted equally for visual recording of color changes and for light scattering measurements at 620 nm using a spectrophotometer (Shimadzu UV-1780). For comparative analysis of BGL enzymatic activities from different species, light scattering at 620 nm was monitored in real time using a microplate reader (BioTek Synergy H1) with four replicates per sample, and curves were plotted with GraphPad Prism 9. Immunoblotting of corresponding proteins was performed using anti-GFP (Roche, 11814460001) and anti-mouse (AlpalifeBio, KTSM1325) antibodies.

For substrate specificity analysis with 14 plant species, each 0.16 g of tissue sample was homogenized in 2 mL water, centrifuged, and 400 μL of supernatant was mixed with either DMSO, 100 μg X-gal (Yeasen, 10901ES03), X-gluc (Yeasen, 10904ES01), or indican. For *Arabidopsis* transgenic genotyping, approximately 2 cm² of leaf tissue was homogenized in 250 μL water, and 100 μL of *Persicaria tinctoria* extract was added to 200 μL of supernatant. The reaction mixtures were incubated at room temperature (25°C), and color changes were observed and documented after 2 h and 24 h using a Canon EOS 6D camera.

### Histochemical detection of PtBGL activity via indican staining

Seedlings were fixed in 90% (v/v) acetone at –20°C for 2 h, washed twice with PBS, and then incubated for 16 h at room temperature (25°C) with gentle shaking in indican staining solution. The staining solution was either crude indican extract from *Persicaria tinctoria* or a solution consisting of 5 mM potassium ferrocyanide, 5 mM potassium ferricyanide, 1 mM magnesium chloride, and 1 mg/mL indican in PBS. Both solutions yielded the same staining results. After staining, samples were washed twice with PBS and cleared with 70% ethanol. The imaging was captured with Zeiss AXIO zoom V16 equipped with AxioCam 305 color.

### Confocal microscopy

Fluorescence signals in tobacco leaves, *Arabidopsis* leaves, and roots were captured using a Zeiss LSM980 confocal laser scanning microscope. Excitation and emission wavelengths were set at 514 nm and 531–551 nm for mCitrine, and 543 nm and 634–704 nm for chlorophyll, respectively.

### Extraction of crude indican from the indigo plant and imprint dyeing

Juvenile and mature leaves of *Persicaria tinctoria* and *Strobilanthes cusia* were collected and subjected to two different treatments, which included microwave heating at 900 W for 2 min or boiling water bath for 10 min. For each 0.1 g of leaf tissue, 1 mL of distilled water was added, and samples were thoroughly homogenized using a mortar or bead mill. The homogenates were centrifuged at 8,000 × g for 5 min, and the resulting supernatants were used for enzymatic assays and indigo staining.

For imprint dyeing, white cotton fabric was soaked in crude indican extract for 2 h at room temperature. The extract was prepared by filtering the homogenates through two layers of cotton fabric. After soaking, the fabric was removed and air-dried. Four- to six-week-old *Arabidopsis* seedlings overexpressing PtBGL were placed on the cotton fabric, which was then folded and gently hammered with a plastic mallet until plant tissues were disrupted. The fabric was incubated under high humidity conditions (>90%) for 24 h, followed by washing three times with mild detergent. The imprinting results were imaged using a Canon EOS 6D camera.

## Results

### Heterologously expressed PtBGL efficiently hydrolyzes indican to produce intense indigo coloration

PtBGL from *Persicaria tinctoria* was localized to chloroplasts by immunocytochemical analysis and catalyzes the hydrolysis of the colorless substrate, indican, originally stored in vacuoles, to release indoxyl and glucose (Minami et al., 1997, 2000; Inoue et al., 2018). Indoxyl is spontaneously oxidized in the presence of oxygen and dimerized to form insoluble blue indigo pigment (**Figure 1A**). To verify whether heterologously expressed PtBGL retains its subcellular localization and catalytic function, we transiently expressed the full-length PtBGL fused with the fluorescent protein mCitrine at its C-terminus in *Nicotiana benthamiana* (tobacco) leaves. Confocal microscopy revealed a strong co-localization between PtBGL-mCitrine fluorescence and chlorophyll autofluorescence, confirming chloroplast targeting of PtBGL in tobacco (**Supplementary Figure S1**). Deletion of the N-terminal 28-amino acid predicted chloroplast transit peptide (ΔTP-PtBGL) abolished chloroplast localization (**Supplementary Figure S1**; Minami et al., 1999), indicating this sequence is essential for proper targeting. Both full-length and truncated PtBGL proteins exhibited robust catalytic activity toward indican (**Figure 1B**; Minami et al., 1999). Incubation of indican with supernatants of tobacco leaf extracts expressing PtBGL led to visible blue coloration within 10 min, intensifying to deep blue after 1 hour (**Figures 1C, D**). Indigo’s poor solubility leads to the formation of suspended particles, allowing turbidity at 620nm to serve as a proxy for product accumulation. A strong correlation with color intensity was observed (**Figures 1C, D**), and this measurement was subsequently used to monitor PtBGL enzymatic activity.

### Indigo formation via the PtBGL–indican system displays high species specificity

**Figure 2:**
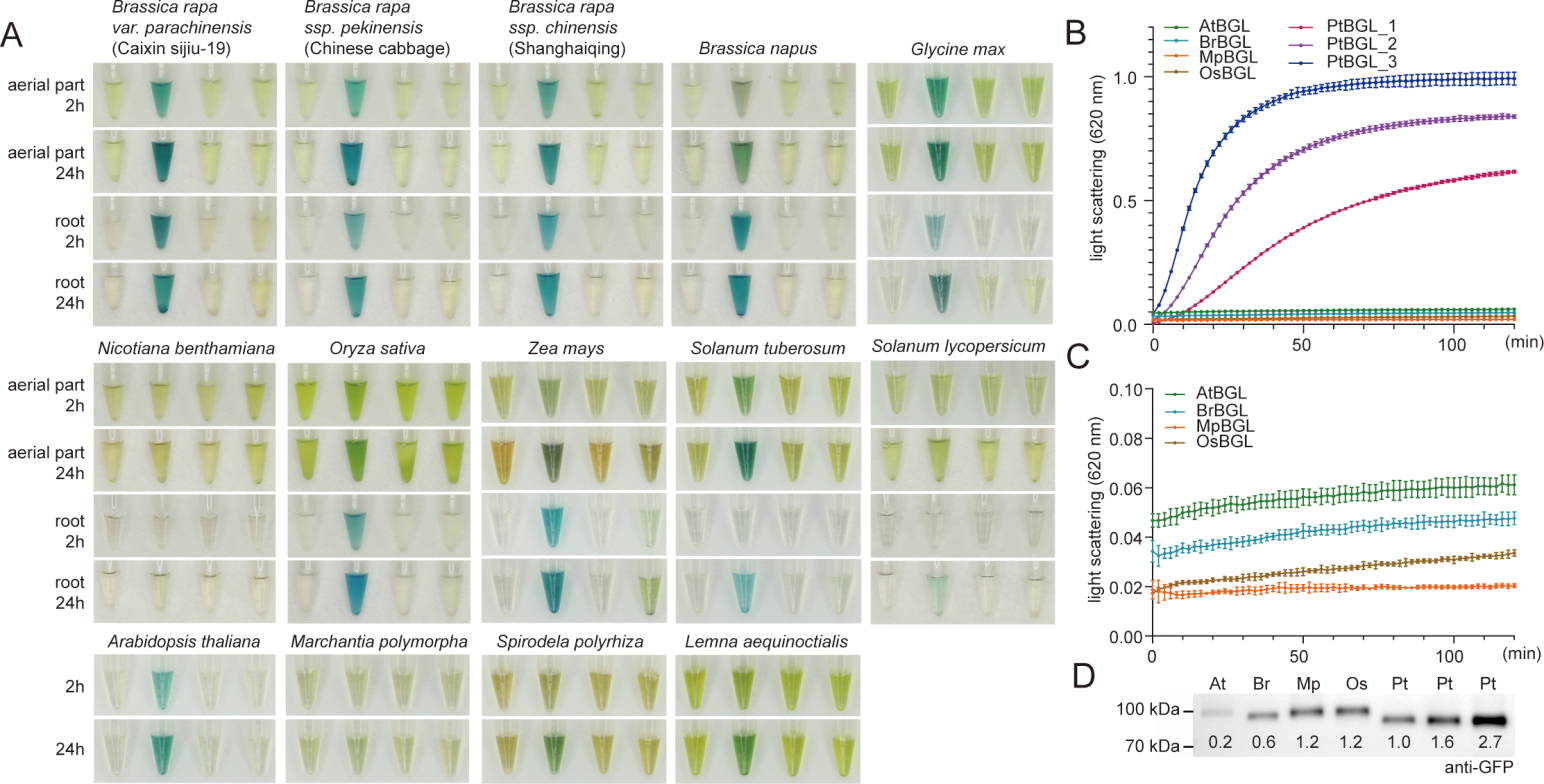
PtBGL–indican reaction exhibits high specificity. **(A)** Most tested species lack endogenous β-glucosidase activity capable of efficiently hydrolyzing indican. Seedlings of *Brassica rapa*, *Brassica napus*, *Glycine max*, *Nicotiana benthamiana*, *Oryza sativa*, *Zea mays*, and *Solanum lycopersicum* were sampled 7 days after germination on MS medium, while *Solanum tuberosum* was collected after 15 days of soil cultivation. For these species, shoots and roots were harvested separately and homogenized. Whole tissues were used for *Arabidopsis thaliana* (10 days on MS medium), *Marchantia polymorpha*, *Spirodela polyrhiza*, and *Lemna aequinoctialis* (15 days after subculture in fresh medium). Soluble protein extracts were incubated with four substrates (left to right): DMSO (negative control), X-gal, X-gluc, and indican. Images were captured at the indicated time points after substrate addition. **(B)** Enzymatic kinetics of PtBGL and orthologous β-glucosidases from *Arabidopsis thaliana* (AtBGL), *Brassica rapa* (BrBGL), *Marchantia polymorpha* (MpBGL), and *Oryza sativa* (OsBGL). PtBGL_1, PtBGL_2, and PtBGL_3 denote the positive control *35S::PtBGL-mCitrine* infiltrated at three different *Agrobacterium* concentrations. The corresponding protein accumulation levels are shown in the last three lanes of the Western blot in panel **(D)** Compared to PtBGL, these homologs exhibited markedly reduced hydrolytic activity toward indican. Data represent mean ± 95% confidence interval; n = 4 per data point. **(C)** Enlarged Y-axis view of panel **(B)** to highlight activity differences among low-activity enzymes. **(D)** Protein levels of lysates used in panels **(B, C)**, analyzed by western blotting using an anti-GFP antibody. Numbers below each band indicate grayscale intensity normalized to the PtBGL_1 sample. Results in panels **(B–D)** are representative of four independent experiments with consistent results.

To assess PtBGL–indican as a reporter compared to LacZ–X-gal and GUS–X-gluc, we tested enzymatic activity in extracts from fourteen commonly used plant species (**Supplementary Figure S2**). Most samples developed strong blue coloration within 2 h when incubated with X-gal, except for tobacco, *Solanum lycopersicum* (tomato), and *Marchantia polymorpha* (**Figure 2A**). In contrast, no visible color development was observed with X-gluc even after 24 h of incubation (**Figure 2A**). With indican, only maize tissues showed faint blue coloration in both aerial and root parts within 2 h, while other species showed no coloration 24 h after incubation (**Figure 2A**), suggesting distinct substrate and species specificity for PtBGL–indican.

To assess whether the limited hydrolysis of indican observed in most species resulted from low endogenous BGL activity at the sampling stage, we evaluated the enzymatic activity of BGL orthologs cloned from representative species. We selected four PtBGL orthologs, each being the gene with the highest protein sequence similarity to PtBGL in *Arabidopsis thaliana* (AtBGL), *Brassica rapa* (BrBGL), *Oryza sativa* (OsBGL), and *Marchantia polymorpha* (MpBGL). They share 42.72% to 52.73% sequence identity with PtBGL. All these BGLs share conserved basal amino acid sequences characteristic of β-glucosidases. Specifically, the conserved catalytic motifs include the TFNEP (Thr-Phe-Asn-Glu-Pro) and ITENG (Ile-Thr-Glu-Asn-Gly) sequences, which contain the catalytic nucleophile (Glu) and acid/base (Glu) residues critical for glycosidic bond cleavage. However, the Phe (F) residue in the TFNEP motif and the Ile (I) and Thr (T) residues in the ITENG motif are not fully conserved across all orthologs, indicating some sequence variability in these positions while retaining the core catalytic functionality (**Supplementary Figure S3C**). Most residues predicted to form the indican-binding pocket in PtBGL were conserved across the orthologs, with His217 being the main exception (**Supplementary Figures S3B, C**). Docking results showed that key substrate-recognition interactions, including hydrogen bonds involving His217 and Glu474 and π-π stacking interactions from Trp389 and Tyr345, were generally preserved (**Supplementary Figure S3**).

**Figure 3:**
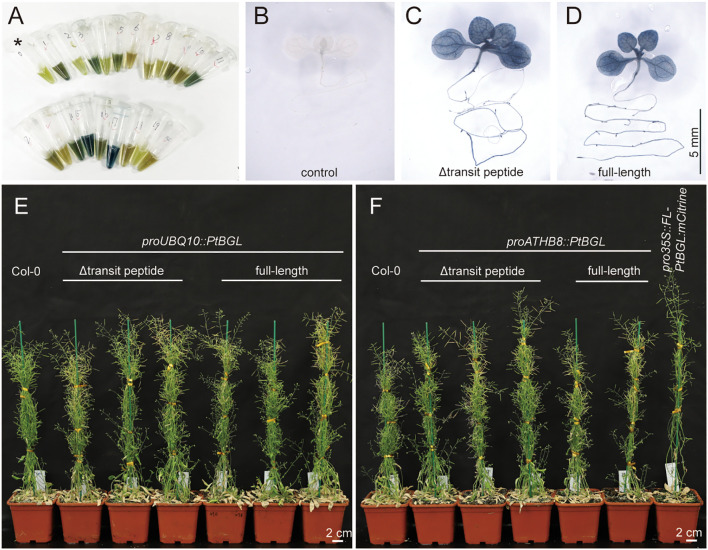
PtBGL–indican system enables genetic screening and gene expression analysis. **(A)** Detection of positive transformants by mixing leaf lysates with boiled *Persicaria tinctoria* leaf extract containing indican. Image was taken 24 h after incubation. Tubes in the upper row correspond to transformant candidates expressing full-length PtBGL; the first tube (marked with an asterisk) is a wild-type control. Tubes in the lower row contain transformants expressing ΔTP-PtBGL. Both PtBGL variants are driven by the UBQ10 promoter. **(B–D)** Native staining in 10-day-old seedlings expressing PtBGL variants under the ATHB8 promoter. Wild-type control seedlings were imaged with shorter exposure for better visualization. **(E)** Most 7-week-old plants expressing ProUBQ10-driven PtBGL variants exhibit no obvious phenotypic abnormalities compared to wild-type (Col-0). **(F)** 7-week-old plants expressing PtBGL variants under the ATHB8 promoter, as well as 35S promoter-driven full-length PtBGL fused to mCitrine, show no visible phenotypic defects.

The C-terminal mCitrine tag did not impair catalytic performance, as untagged PtBGL (**Figure 1C**) and PtBGL-mCitrine (**Figure 2B**) exhibited virtually identical staining kinetics. Accordingly, all four β-glucosidases were tagged with mCitrine and transiently expressed in tobacco leaves under the control of the CaMV 35S promoter. To account for potential expression differences, a reference activity gradient was established using PtBGL expressed at varying *Agrobacterium* concentrations. Protein expression levels were verified by anti-GFP western blotting. Although the key substrate-binding residues were largely conserved among PtBGL and its orthologs, their catalytic activities toward indican differed markedly. Enzymatic assays showed that OsBGL exhibited only weak activity, while MpBGL showed no detectable activity (**Figures 2B, C**). AtBGL and BrBGL consistently accumulated at relatively low levels, which precluded an accurate assessment of their catalytic efficiencies (**Figures 2B–D**). Nevertheless, subsequent staining experiments in *Arabidopsis*, presented in the following results, demonstrated the absence of endogenous background, indicating that AtBGLs do not interfere with indican hydrolysis under the tested conditions (**Figures 3A, B**). Whether BrBGLs exhibit similarly minimal background activity in *Brassica rapa* remains to be determined through stage-specific evaluation. Together with the low background observed across most species tested, these results support the utility of PtBGL as a specific and reliable reporter, particularly at the seedling stage.

### PtBGL can serve as a reporter for transgenic selection and gene expression pattern analysis

An ideal reporter gene should allow high expression without adversely affecting plant growth. To evaluate the impact of PtBGL expression on development, full-length and truncated PtBGL were constitutively expressed in *Arabidopsis* under a strong promoter ProUBQ10. T1 transgenic seedlings were rapidly confirmed by visible blue coloration after treating leaf extract supernatants with indican (**Figure 3A**). This allowed for efficient screening of positive lines. Expression of full-length or truncated PtBGL did not affect plant growth in most lines. The majority of T3 progeny developed normally and were phenotypically indistinguishable from wild-type plants (**Figure 3E**). However, a small subset of lines (3 out of 48) with relatively high levels of truncated PtBGL expression exhibited growth defects, including leaf curling, dwarfism, and reduced fertility (**Supplementary Figure S4A**). These results suggest that PtBGL, particularly the full-length version, enables efficient transgenic screening and is generally well tolerated by plants when expressed at detectable levels.

To evaluate its application for gene expression pattern analysis, *PtBGL* was driven by the promoter of *AtHB8*, which is mainly expressed in the vascular tissues (Scarpella et al., 2004). Both full-length and truncated variants produced comparable staining patterns, and transgenic plants did not exhibit altered phenotypes compared to wild-type (**Figure 3F**). Despite successful staining, the blue coloration produced by the PtBGL–indican system is relatively dark (**Figures 3B–D**). Although the staining advantage over GUS is limited in small samples such as *Arabidopsis*, the low cost and simplicity of the indican substrate make PtBGL a promising reporter for larger tissue samples.

Additionally, subcellular localization was examined by constitutively expressing full-length PtBGL fused to mCitrine under the 35S promoter, which also did not produce any detectable growth abnormalities (**Figure 3F**). The fusion protein localized predominantly to chloroplasts in leaves and leucoplasts in roots, consistent with results from transient expression in tobacco (**Supplementary Figures S4B, C**).

### Heat treatment of indigo plants provides an easy and low-cost source of indican substrate

Chemically synthesized indican costs approximately 800 USD per gram, which is relatively less expensive than X-gluc (approximately 1600 USD per gram), but still not affordable for large-scale applications. In indigo-producing plants such as *Persicaria tinctoria*, *Isatis indigotica*, *Isatis tinctoria*, and *Strobilanthes cusia*, indican accumulates abundantly in the vacuoles of leaf cells and is spatially separated from endogenous BGL. Heating the leaves rapidly denatures BGL, enabling straightforward preparation of crude extracts enriched in intact indican (Jiang et al., 2022; Hu et al., 2023). We treated young and old leaves of *Persicaria tinctoria* and *Isatis indigotica* using boiling water or microwave heating (**Supplementary Figures S5A, B**), and the resulting crude extracts exhibited high staining efficiency when incubated with tobacco lysate expressing PtBGL (**Supplementary Figure S5C**). Among the tested samples, boiled young leaves of *Persicaria tinctoria* produced the fastest and most distinct blue color development, with minimal chlorophyll background interference. Indigo plants are inexpensive, costing approximately 4 USD per kilogram of fresh stems and leaves, which yield about 1 liter of indican-rich crude extract. This volume is sufficient for over 5,000 transgenic screening reactions or approximately 40 whole-plant gene expression assays in mature *Arabidopsis* plants. Since *Persicaria tinctoria* can be readily propagated from seeds, the PtBGL–indican system offers an economical, efficient, and scalable substrate source for plant molecular biology applications.

### PtBGL transgenic plants enable local indican catalysis to improve dye durability in plant imprinting

**Figure 4:**
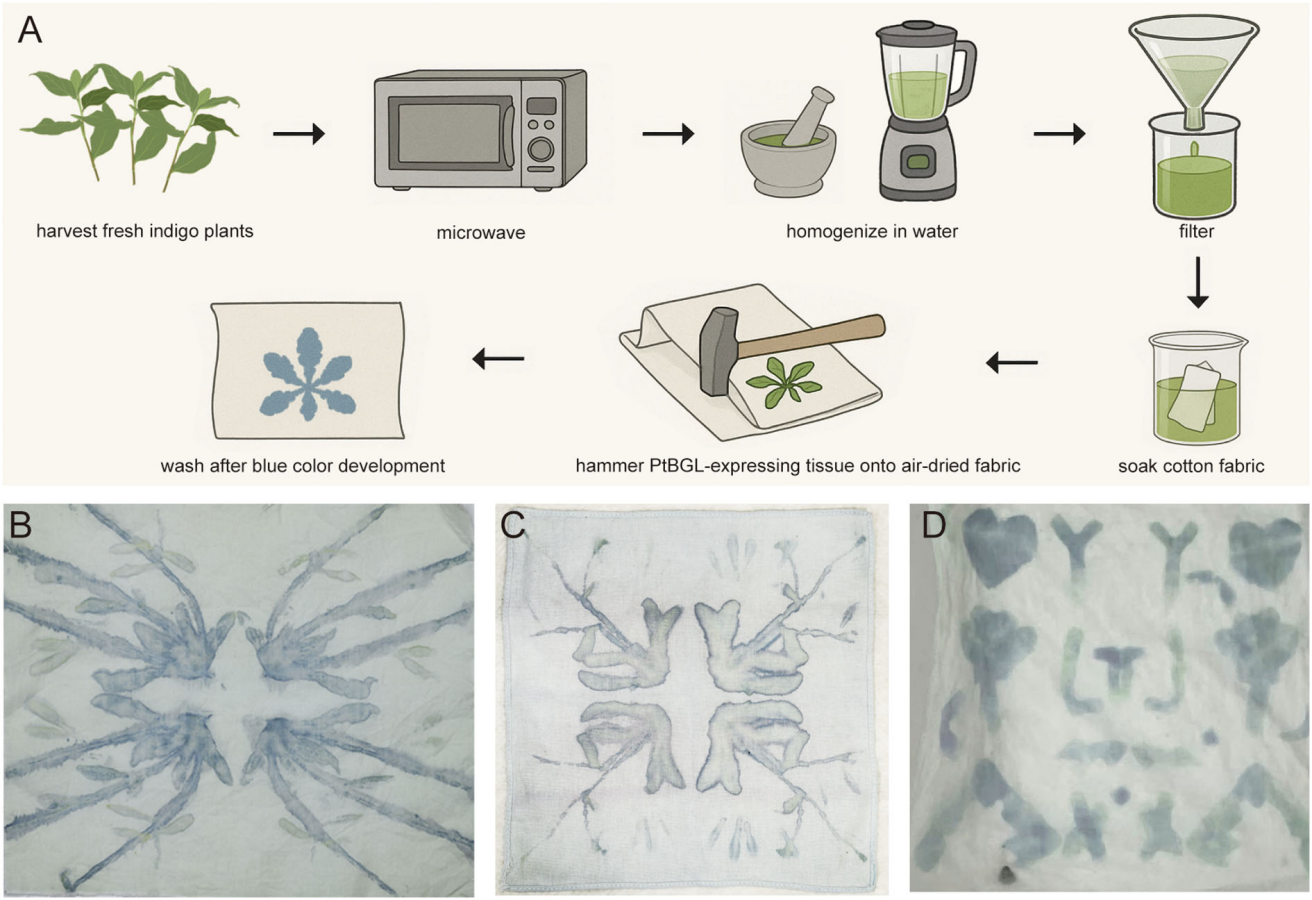
Heterologous expression of PtBGL enables enzymatic blue dye formation and plant-based fabric imprinting. **(A)** Schematic workflow of fabric imprinting using PtBGL-expressing plant material. *Persicaria tinctoria* leaves were harvested for indican extraction, subjected to microwave treatment, and homogenized using a mortar or blender. The crude extract was filtered, and cotton fabric was immersed in the filtrate for 2 h, followed by air drying. Transgenic *Arabidopsis* leaves expressing full-length PtBGL were applied onto the pretreated fabric, enabling enzymatic hydrolysis of indican and subsequent indigo formation. The resulting blue imprints remained visible and stable after three washes with tap water containing laundry detergent. **(B)** Five-week-old *Arabidopsis* plants expressing full-length PtBGL were pressed onto cotton fabric folded twice (yielding four layers). **(C)** Four-week-old *Arabidopsis* plants expressing ΔTP-PtBGL were printed onto fabric folded twice (four layers). **(D)** Tobacco leaves transiently expressing full-length PtBGL were excised and trimmed into defined shapes prior to printing onto fabric folded once (two layers). Imprints in panels **(B–D)** are after three washes with tap water containing a laundry detergent.

Building on the efficient and scalable preparation of indican crude extracts for molecular biology applications, we explored the practical utility of this system in plant-based textile imprinting. Conventional dyeing techniques frequently encounter challenges with color fading due to the water solubility and limited fiber affinity of natural pigments, such as anthocyanins, even after chemical fixation. In contrast, indigo precipitates within textile fibers, exhibiting exceptional wash fastness. Traditional indigo dyeing relies on environmentally detrimental strong alkaline reduction processes. However, Hsu et al. developed a sustainable alternative by applying soluble indican to textile fibers, followed by treatment with BGL, which catalyzes the localized conversion of indican into indigo, enabling eco-friendly coloration (Hsu et al., 2018). Employing this method, we immersed fabrics in indican crude extracts to enhance substrate penetration into fibers, followed by air drying. Subsequently, plants overexpressing PtBGL were pressed onto the treated fabrics, where PtBGL catalyzed the hydrolysis of indican into indigo, producing distinct and durable blue patterns (**Figure 4A**). These imprints exhibited robust resistance to multiple wash cycles, confirming superior water fastness (**Figures 4B–D**).

## Discussion

In this study, we established a reporter gene system based on *Persicaria tinctoria* β-glucosidase and its natural substrate, indican, providing a cost-effective and plant-compatible alternative to conventional systems. When expressed in *Arabidopsis* and tobacco, PtBGL exhibited strong enzymatic activity and localized to plastids. This system enabled efficient transgenic screening and expression analysis with minimal background staining, demonstrating its utility in common model species. Given that β-glucosidases are widely distributed in plants, potential interference from endogenous activities may have discouraged their exploration as reporter systems. Our preliminary observations in 14 species indicate that PtBGL can be applied across a broad range of plants, underscoring its potential as a versatile tool in plant molecular biology.

A significant advantage of the PtBGL–indican system is the low cost of its substrate relative to that used in the GUS–X-gluc system. While the system shows no significant advantage over GUS for analyzing gene expression patterns in small tissues, it offers considerable benefits for studying spatial and temporal expression in large organs, such as leaves and stems of crops like cabbage, soybean, and tomato, and is also well-suited for high-throughput screening. The blue pigment produced from indican typically appears grayer and less saturated than that from X-gluc, potentially limiting contrast in some tissues. However, this limitation can be addressed through halogenation of the indole ring. Microbial biosynthetic strategies have been explored to diversify indigoid pigments, which offer potential routes to expand the chromogenic capacity of the PtBGL–indican system. Tryptophan halogenases, when introduced into *E. coli*, can generate halogenated tryptophan derivatives, which are subsequently converted into a spectrum of colored products through tryptophanase (TnaA) and flavin-dependent monooxygenase (FMO) (Hsu et al., 2018; Lee et al., 2021). Co-expression of these enzymes with *Persicaria tinctoria* glucosyltransferase (PtUGT1) enables the production of halogenated indican derivatives directly from tryptophan. These compounds yield hydrolysis products with varied and more vibrant colors, providing opportunities to enhance both the visual impact and specificity of the reporter system. Another potential limitation of the PtBGL–indican system is its incompatibility with plant species that endogenously synthesize indican or closely related indoxyl glycosides and express active β-glucosidases (e.g., *Indigofera tinctoria*, *Isatis tinctoria* and certain *Wrightia* and *Lonchocarpus* species). In these taxa, constitutive precursor accumulation and endogenous β-glucosidase activity would cause high background staining. Researchers planning to use PtBGL-indican system in species or tissues not tested in this study are therefore recommended to assess background activity prior to application.

To improve system accessibility, we applied a simple protocol to extract crude indican from indigo-producing plants using heat-based methods such as boiling and microwave treatment (Jiang et al., 2022; Hu et al., 2023). These procedures inactivated endogenous β-glucosidases, thereby preserving the stability of the substrate for subsequent enzymatic assays or tissue imprinting applications. Interestingly, this approach is conceptually similar to a traditional dyeing method “half-boiled and half-raw” recorded in Tiangong Kaiwu (The Exploitation of the Works of Nature, circa 1637), where thermally treated plant extracts rich in soluble indican were mixed with fermented materials containing active enzymes. The combination allowed indican to infiltrate textile fibers, while enzymatic activity in the fermented portion triggered pigment formation within the fabric (Hsu et al., 2018). However, a limitation of this approach in imprinting dyeing is that the activity of PtBGL is not sufficiently high, which requires extended reaction times. During this period, soluble PtBGL, indican and released indoxyl diffuse and volatilize toward the fabric edges, resulting in a coffee-ring effect. This challenge could be addressed through enzyme engineering to enhance PtBGL activity, enabling rapid indigo formation upon cell disruption for more uniform dyeing. Nevertheless, this form of imprinting is visually distinctive, low-cost, and free from synthetic dyes or expensive reagents. As such, it offers a compelling platform for educational activities in biology classrooms and public engagement workshops.

## Data Availability

The original contributions presented in the study are included in the article/**Supplementary Material**. Further inquiries can be directed to the corresponding authors.

## Supplementary material

Supplementary Figure 1 Subcellular localization of mCitrine-tagged PtBGL variants in tobacco leaves. Full-length PtBGL (containing the native transit peptide) localizes to chloroplasts, whereas the truncated variant (Δ1–28 aa, transit peptide deleted) fails to target chloroplasts. Images (from left to right) show mCitrine fluorescence (pseudocolored green), chlorophyll autofluorescence (red, chloroplast marker), and merged channels. The scale bar applies to all panels. Data are representative of observations in at least 10 cells from two independent experiments.

Supplementary Figure 2 Growth conditions of the samples used for the experiments shown in **Figure 2**. All scale bars represent 1 cm.

Supplementary Figure 3 Structural modeling and predicted binding interactions of PtBGL with indican. **(A)** The 3D structure of the PtBGL catalytic domain (residues Lys35–Asn511) predicted by AlphaFold3, with indican docked using AutoDock. **(B)** Predicted key residues mediating substrate binding: His217 and Glu474 form hydrogen bonds with the glucose moiety of indican, whereas the indole ring of indican is stabilized by π-π stacking interactions with the aromatic side chains of Trp389 and Tyr345. **(C)** Sequence alignment of the five β-glucosidases evaluated in the catalytic activity assays. Four residues implicated in indican binding are indicated by red asterisks; among them, His217 is the sole residue showing variability across the sequences. β-glucosidase-specific TFNEP and ITENG motifs, containing catalytic Glu residues, are highlighted in red boxes.

Supplementary Figure 4 Phenotypic effects and subcellular localization of PtBGL in *Arabidopsis*. **(A)** Overexpression of truncated PtBGL under the constitutive ProUBQ10 promoter occasionally led to developmental abnormalities. Among 48 independent transgenic lines, three exhibited dwarfism, upward-curved rosette leaves, and male sterility. **(B)** Subcellular localization of PtBGL-mCitrine in *Arabidopsis* leaves, showing chloroplast-associated fluorescence similar to that observed in transiently expressed tobacco leaves. Scale bar applies to all panels in **(B)**. **(C)** PtBGL-mCitrine localizes to plastids in *Arabidopsis* root cells.

Supplementary Figure 5 Effects of different heat treatments on indican yield from crude leaf extracts. **(A)***Persicaria tinctoria* leaves used for indican extraction. Two or three light-green juvenile leaves (marked with red dotted lines) and fully expanded dark green mature leaves were collected separately. **(B)***Strobilanthes cusia* leaves used for indican extraction. Juvenile leaves (smaller, light-green leaves marked with red dotted lines) and mature leaves were collected separately. **(C)** Equal weights of juvenile and mature leaves were subjected to microwave heating or water bath boiling to rapidly inactivate endogenous β-glucosidases before extraction. Extracts were then mixed with equal volumes of tobacco lysate expressing full-length PtBGL (+) or control lysate (-), and indican content was estimated by the intensity of blue coloration. The upper panel shows reactions from *Persicaria tinctoria* leaves, and the lower panel shows those from *Strobilanthes cusia*. Juvenile leaves consistently gave stronger blue coloration, indicating higher indican levels.

Supplementary Table 1 Primers used in this study.
