# Supplementary material for: PtBGL: a cost-effective alternative to GUS reporter with applications in plant imprint dyeing
Source: Front Plant Sci. 2026 Jan 6;16:1705524. doi: 10.3389/fpls.2025.1705524 (PMC12819836; doi:10.3389/fpls.2025.1705524)
Supplement: Supplementary Figure 1 — Subcellular localization of mCitrine-tagged PtBGL variants in tobacco leaves. Full-length PtBGL (containing the native transit peptide) localizes to chloroplasts, whereas the truncated variant (Δ1–28 aa, transit peptide deleted) fails to target chloroplasts. Images (from left to right) show mCitrine fluorescence (pseudocolored green), chlorophyll autofluorescence (red, chloroplast marker), and merged channels. The scale bar applies to all panels. Data are representative of observations in at least 10 cells from two independent experiments. [file DataSheet1.pdf]

Supplementary Figure S1

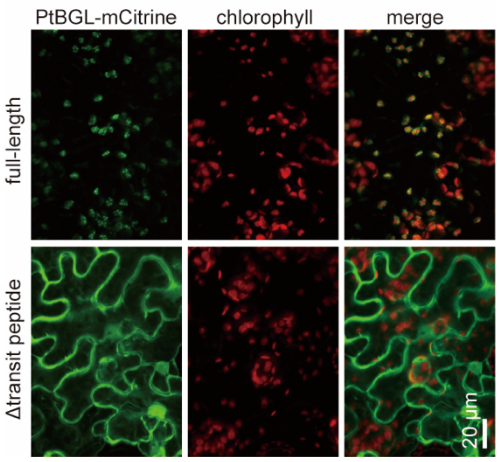

Supplementary Figure S1. Subcellular localization of mCitrine-tagged PtBGL variants in tobacco leaves. Full-length PtBGL (containing the native transit peptide) localizes to chloroplasts, whereas the truncated variant (Δ1–28 aa, transit peptide deleted) fails to target chloroplasts. Images (from left to right) show mCitrine fluorescence (pseudocolored green), chlorophyll autofluorescence (red, chloroplast marker), and merged channels. The scale bar applies to all panels. Data are representative of observations in at least 10 cells from two independent experiments.

Supplementary Figure S2

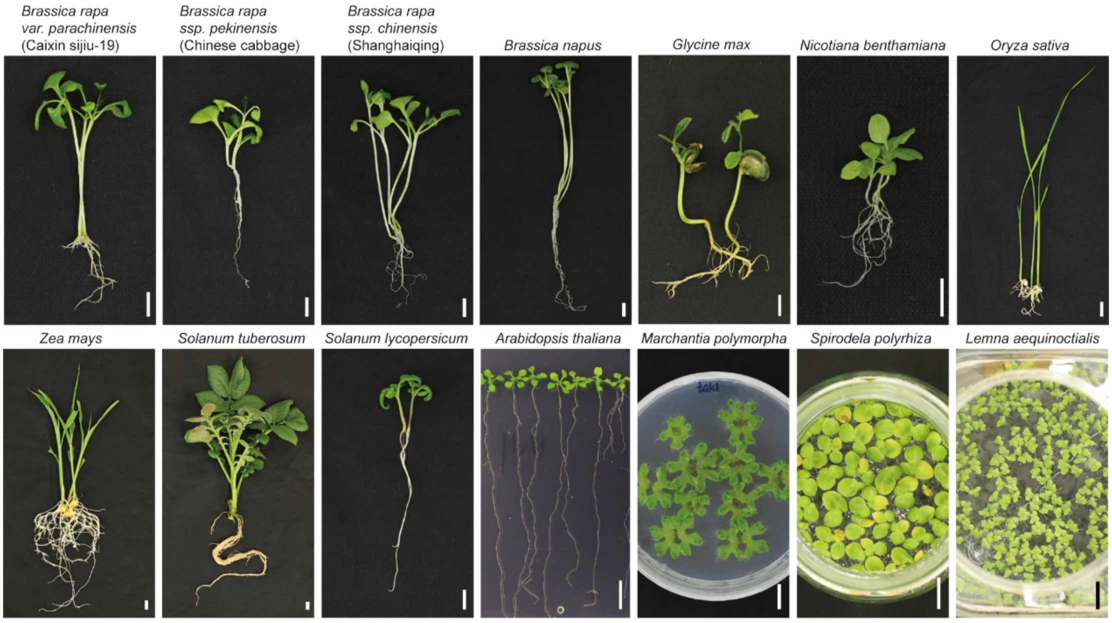

Supplementary Figure S2. Growth conditions of the samples used for the experiments shown in Figure 2. All scale bars represent 1 cm.

Supplementary Figure S3

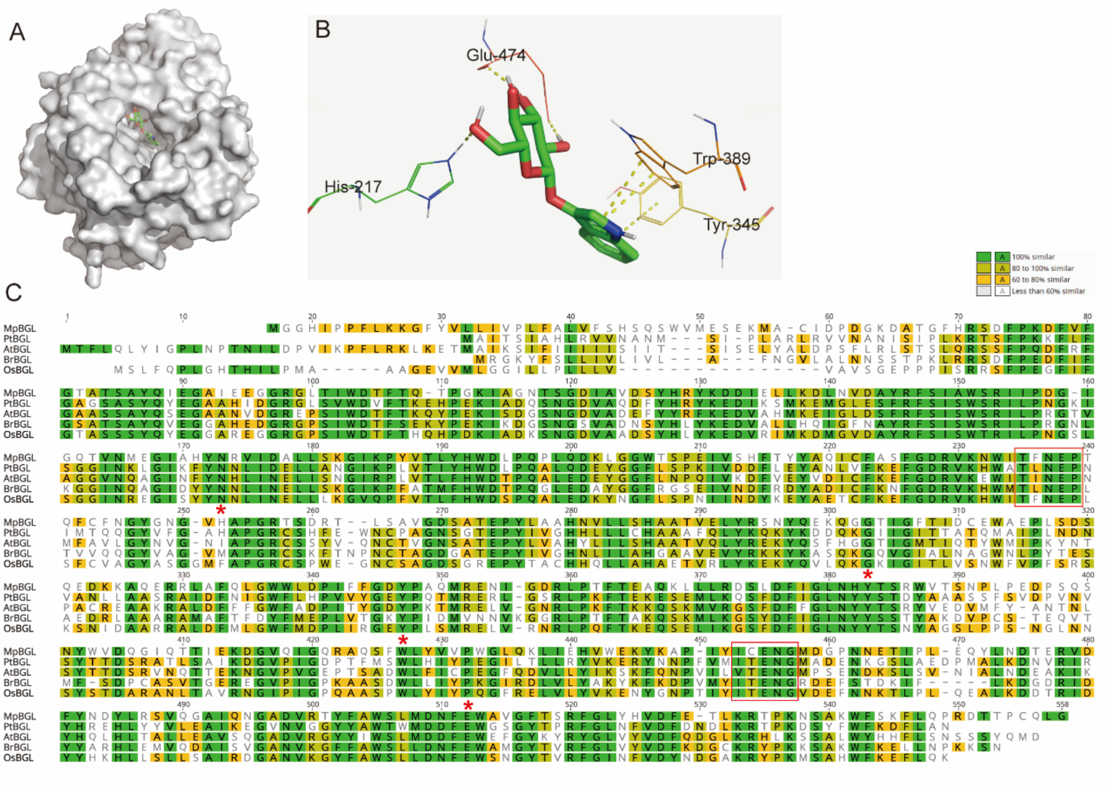

Supplementary Figure S3. Structural modeling and predicted binding interactions of PtBGL with indican. (A) The 3D structure of the PtBGL catalytic domain (residues Lys35–Asn511) predicted by AlphaFold3, with indican docked using AutoDock. (B) Predicted key residues mediating substrate binding: His217 and Glu474 form hydrogen bonds with the glucose moiety of indican, whereas the indole ring of indican is stabilized by π-π stacking interactions with the aromatic side chains of Trp389 and Tyr345. (C) Sequence alignment of the five β-glucosidases evaluated in the catalytic activity assays. Four residues implicated in indican binding are indicated by red asterisks; among them, His217 is the sole residue showing variability across the sequences. β-glucosidase-specific TFNEP and ITENG motifs, containing catalytic Glu residues, are highlighted in red boxes.

Supplementary_Figure_S4

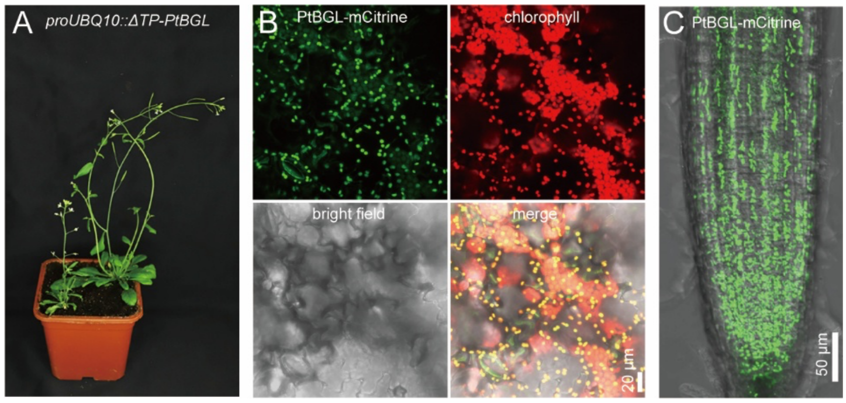

Supplementary Figure S4. Phenotypic effects and subcellular localization of PtBGL in *Arabidopsis*. (A) Overexpression of truncated PtBGL under the constitutive ProUBQ10 promoter occasionally led to developmental abnormalities. Among 48 independent transgenic lines, three exhibited dwarfism, upward-curved rosette leaves, and male sterility. (B) Subcellular localization of PtBGL-mCitrine in *Arabidopsis* leaves, showing chloroplast-associated fluorescence similar to that observed in transiently expressed tobacco leaves. Scale bar applies to all panels in (B). (C) PtBGL-mCitrine localizes to plastids in *Arabidopsis* root cells.

Supplementary_Figure_S5

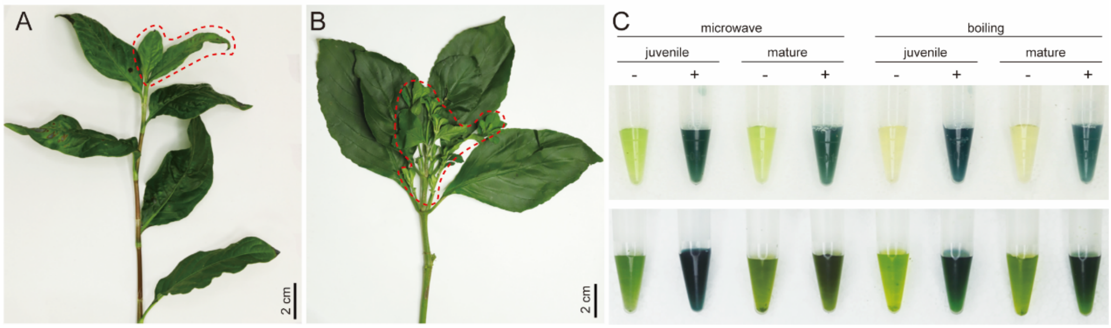

Supplementary Figure S5. Effects of different heat treatments on indican yield from crude leaf extracts. (A) *Persicaria tinctoria* leaves used for indican extraction. Two or three light-green juvenile leaves (marked with red dotted lines) and fully expanded dark green mature leaves were collected separately. (B) *Strobilanthes cusia* leaves used for indican extraction. Juvenile leaves (smaller, light-green leaves marked with red dotted lines) and mature leaves were collected separately. (C) Equal weights of juvenile and mature leaves were subjected to microwave heating or water bath boiling to rapidly inactivate endogenous β-glucosidases before extraction. Extracts were then mixed with equal volumes of tobacco lysate expressing full-length PtBGL (+) or control lysate (-), and indican content was estimated by the intensity of blue coloration. The upper panel shows reactions from *Persicaria tinctoria* leaves, and the lower panel shows those from *Strobilanthes cusia*. Juvenile leaves consistently gave stronger blue coloration, indicating higher indican levels.

Supplementary Table 1. Primers used in this study

primer names	sequences
GGC-PtBGL-FL-FP1	aacaGGTCTCaggctcaATGGCGATCACCTCCATAGC
GGC-PtBGL-FL-RP1*	ACAAAGTTGAGTCCGAATCGAGG
GGC-PtBGL-FL-FP2*	CCTCGATTCGGACTCAACTTTGT
GGC-PtBGL-FL-RP2	aacaGGTCTCtctgaATTTGCAAGGAAGTCCTTGAAC
GGC-PtBGL-dTP-FP**	aacaGGTCTCaggctCAATGAACATAAGCATTCCGCTTAAGC
GGC-PtBGL-dTP-RP**	aacaGGTCTCtctgaATTTGCAAGGAAGTCCTTGAAC
GGA-proATHB8-FP	aacaGGTCTCaacctcaggaatgatgattgacgataatg
GGA-proATHB8-RP	aacaGGTCTCttgttctttgatcctctccgatctctc
1301-mCitrine-NcoI-FP	CGGGGGACTCTTGACCatggccctagggTCAGGGGCAGGAGCGGGCA
1301-mCitrine-Eco91I-RP	GGGAAATTCGAGCTGGTCACCttaCTTGTACAGCTCGTCCAT
1301-AtBGL-mCit-FP	cGGGGGACTCTTGACCatgggaATGACTTTTCTTCAATTATATATAGGGCC
1301-AtBGL-mCit-RP	CTCCTGCCCCTGAccctaggccGTCCATTTGATAGGAGGATGAG
1301-BrBGL-mCit-FP	cGGGGGACTCTTGACCatgggaATGAGAGGAAAATATTTTTCTTTACTAATAG
1301-BrBGL-mCit-RP	CTCCTGCCCCTGAccctaggccATTGCTTTTCTTTGGATTCAACAAC
1301-MpBGL-mCit-FP	cGGGGGACTCTTGACCatgggaATGGGTGGGCACATTCCTC
1301-MpBGL-mCit-RP	CTCCTGCCCCTGAccctaggccACCTAATTGGCAAGGTGTGGTG
1301-OsBGL-mCit-FP	cGGGGGACTCTTGACCatgggaATGTCTCTCTTTCAGCCTCTG
1301-OsBGL-mCit-RP	CTCCTGCCCCTGAccctaggccTTTCTGGAGGAACTCCTTGAAC
1301-PtBGL-mCit-FP	cGGGGGACTCTTGACCatgggaATGGCGATCACCTCCATAGC
1301-PtBGL-mCit-RP	CTCCTGCCCCTGAccctaggccATTTGCAAGGAAGTCCTTGAAC

*, Primers for mutating the internal *Bsa* I site

**, Primers for cloning the PtBGL variant lacking transit peptide
